# 1-(2-Bromo­phen­yl)-3-(4-chloro­butano­yl)thio­urea

**DOI:** 10.1107/S160053681201327X

**Published:** 2012-03-31

**Authors:** Mohd Sukeri Mohd Yusof, Nur Farhana Embong, Suhana Arshad, Ibrahim Abdul Razak

**Affiliations:** a Department of Chemical Sciences, Faculty of Science and Technology, Universiti Malaysia Terengganu, Mengabang Telipot, 21030 Kuala Terengganu, Malaysia; b School of Physics, Universiti Sains Malaysia, 11800 USM, Penang, Malaysia

## Abstract

The asymmetric unit of the title compound, C_11_H_12_BrClN_2_OS, consists of two crystallographically independent mol­ecules. In each mol­ecule, the butano­ylthio­urea unit is nearly planar, with maximum deviations of 0.1292 (19) and 0.3352 (18) Å from the mean plane defined by nine non-H atoms, and is twisted relative to the terminal benzene ring with dihedral angles of 69.26 (7) and 82.41 (7)°. An intra­molecular N—H⋯O hydrogen bond generates an *S*(6) ring motif in each butano­ylthio­urea unit. In the crystal, N—H⋯O hydrogen bonds link the two independent mol­ecules together, forming an *R*_2_^2^(12) ring motif. The mol­ecules are further connected into a tape along the *c* axis *via* N—H⋯S and C—H⋯S hydrogen bonds.

## Related literature

For related structures, see: Binzet *et al.* (2009 ▶); Khawar Rauf *et al.* (2006 ▶); Shoukat *et al.* (2007 ▶); Yesilkaynak *et al.* (2010 ▶); Yusof *et al.* (2007 ▶). For hydrogen-bond motifs, see: Bernstein *et al.* (1995 ▶). For stability of the temperature controller used in the data collection, see: Cosier & Glazer (1986 ▶).
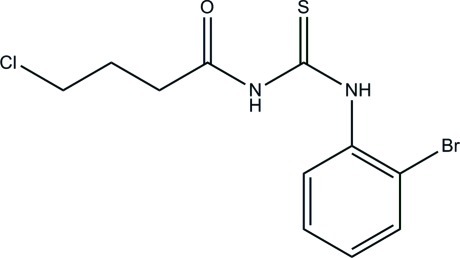


## Experimental

### 

#### Crystal data

C_11_H_12_BrClN_2_OS
*M* *_r_* = 335.65
Monoclinic, 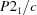

*a* = 14.1384 (2) Å
*b* = 11.1948 (1) Å
*c* = 17.7264 (2) Å
β = 107.955 (1)°
*V* = 2669.03 (5) Å^3^
*Z* = 8
Mo *K*α radiation
μ = 3.42 mm^−1^
*T* = 100 K
0.39 × 0.17 × 0.11 mm

#### Data collection

Bruker SMART APEXII CCD area-detector diffractometer
Absorption correction: multi-scan (*SADABS*; Bruker, 2009 ▶) *T*_min_ = 0.350, *T*_max_ = 0.696
34224 measured reflections
8749 independent reflections
6599 reflections with *I* > 2σ(*I*)
*R*_int_ = 0.029

#### Refinement

*R*[*F* ^2^ > 2σ(*F* ^2^)] = 0.031
*wR*(*F* ^2^) = 0.067
*S* = 1.02
8749 reflections
307 parameters
H-atom parameters constrained
Δρ_max_ = 0.64 e Å^−3^
Δρ_min_ = −0.48 e Å^−3^

### 

Data collection: *APEX2* (Bruker, 2009 ▶); cell refinement: *SAINT* (Bruker, 2009 ▶); data reduction: *SAINT*; program(s) used to solve structure: *SHELXTL* (Sheldrick, 2008 ▶); program(s) used to refine structure: *SHELXTL*; molecular graphics: *SHELXTL*; software used to prepare material for publication: *SHELXTL* and *PLATON* (Spek, 2009 ▶).

## Supplementary Material

Crystal structure: contains datablock(s) global, I. DOI: 10.1107/S160053681201327X/is5098sup1.cif

Structure factors: contains datablock(s) I. DOI: 10.1107/S160053681201327X/is5098Isup2.hkl

Supplementary material file. DOI: 10.1107/S160053681201327X/is5098Isup3.cml

Additional supplementary materials:  crystallographic information; 3D view; checkCIF report

## Figures and Tables

**Table 1:** Hydrogen-bond geometry (Å, °)

*D*—H⋯*A*	*D*—H	H⋯*A*	*D*⋯*A*	*D*—H⋯*A*
N1*A*—H1*NA*⋯O1*A*	0.84	2.01	2.6847 (19)	136
N1*B*—H1*NB*⋯O1*B*	0.84	1.97	2.6464 (19)	136
N1*A*—H1*NA*⋯O1*B*	0.84	2.33	2.9976 (18)	137
N1*B*—H1*NB*⋯O1*A*	0.84	2.39	3.0566 (19)	137
N2*A*—H2*NA*⋯S1*B*^i^	0.85	2.56	3.3931 (15)	168
N2*B*—H2*NB*⋯S1*A*^ii^	0.84	2.56	3.3928 (14)	171
C9*B*—H9*BA*⋯S1*A*^ii^	0.99	2.87	3.7237 (18)	145
C9*B*—H9*BB*⋯S1*B*^iii^	0.99	2.84	3.7248 (18)	149

Symmetry codes: (i) 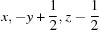; (ii) 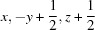; (iii) 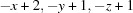.

**Fig. 1.**
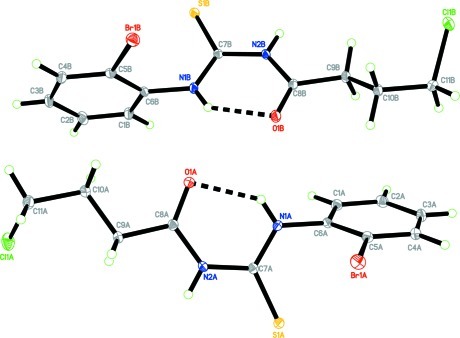
The molecular structure of the title compound with atom labels with 30% probability displacement ellipsoids.

**Fig. 2.**
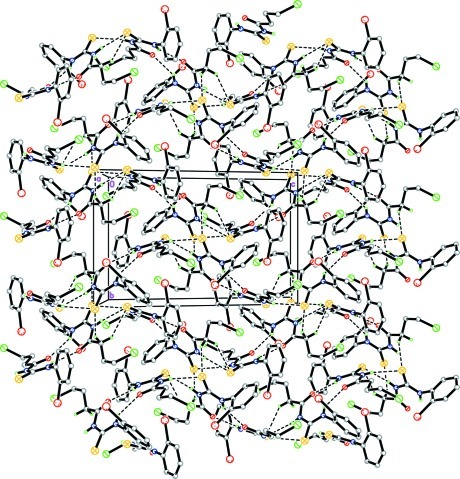
The crystal packing of the title compound. The H atoms not involved in the intermolecular interactions (dashed lines) have been omitted for clarity.

## supplementary crystallographic information

### Comment

Thiourea derivatives are flexible ligand and able to coordinate to the metal centre as mono-dentat or multi-dentat depended on the substituent group attached to the both of nitrogen atoms (Binzet *et al.*, 2009; Yesilkaynak *et al.*, 2010).

The asymmetric unit of the title compound (Fig. 1), consists of two crystallographically independent molecules *A* and *B*. In both molecules, the intramolecular N1A—H1NA···O1A and N1B—H1NB···O1B hydrogen bonds (Table 1) generate *S*(6) ring motifs (Bernstein *et al.*, 1995). The chlorobutanoylthiourea groups (Cl1A/S1A/O1A/N1A/N2A/C7A–C11A & Cl1B/S1B/O1B/N1B/N2B/C7B–C11B) are twisted about C10A–C11A bond with C9A–C10A–C11A–Cl1A torsion angle of -66.74 (19)° and about C10B–C11B bond with C9B–C10B–C11B–Cl1B torsion angle of 60.18 (19)°, respectively. However, the butanoylthiourea groups (S1A/O1A/N1A/N2A/C7A–C11A & S1B/O1B/N1B/N2B/C7B–C11B) are nearly planar with maximum deviations of 0.1292 (19) Å at atom C10A and 0.3352 (18) Å at atom C10B. The mean plane through the butanoylthiourea group of molecule *A* (S1A/O1A/N1A/N2A/C7A–C11A) makes a dihedral angle of 69.26 (7)° with the terminal benzene ring (C1A–C6A). In molecule *B*, the corresponding value is 82.41 (7)°. The bond lengths and angles are within normal ranges and are comparable to the related structures (Shoukat *et al.*, 2007; Khawar Rauf *et al.*, 2006; Yusof *et al.*, 2007).

In the crystal packing (Fig. 2), intermolecular N—H···O hydrogen bonds (Table 1), form *R*^2^_2_(12) (Bernstein *et al.*,1995) ring motifs. The molecules are further connected into a molecular tape along the *c* axis *via* intermolecular N—H···S and C—H···S hydrogen bonds (Table 1).

### Experimental

An equimolar amount of 2-bromoaniline (1.22 g, 7.09 mmol) in 20 ml acetone was added drop-wise into a stirring acetone solution (75 ml) containing 4-chlorobutanoylchloride (1.00 g, 7.09 mmol) and ammonium thiocyanate (0.54 g, 7.09 mmol). The mixture was refluxed for 1 h. Then, the solution was filtered-off and left to evaporate at room temperature.

### Refinement

N-bound H atoms were located in a difference Fourier map and were fixed at their found locations using riding model with *U*_iso_(H) = 1.2 *U*_eq_(N) (N—H = 0.8391–0.8465 Å). The remaining H atoms were positioned geometrically (C—H = 0.95 or 0.99 Å) and refined using a riding model with *U*_iso_(H) = 1.2 *U*_eq_(C). Five outliners 11 1 15, 6 15 1, 10 1 13, 4 0 6 and 8 0 10 were omitted in the final refinement.

### Crystal data

**Table:**

C_11_H_12_BrClN_2_OS	*F*(000) = 1344
*M**_r_* = 335.65	*D*_x_ = 1.671 Mg m^−^^3^
Monoclinic, *P*2_1_/*c*	Mo *K*α radiation, λ = 0.71073 Å
Hall symbol: -P 2ybc	Cell parameters from 9962 reflections
*a* = 14.1384 (2) Å	θ = 2.4–31.2°
*b* = 11.1948 (1) Å	µ = 3.42 mm^−^^1^
*c* = 17.7264 (2) Å	*T* = 100 K
β = 107.955 (1)°	Block, colourless
*V* = 2669.03 (5) Å^3^	0.39 × 0.17 × 0.11 mm
*Z* = 8	

### Data collection

**Table:**

Bruker SMART APEXII CCD area-detector diffractometer	8749 independent reflections
Radiation source: fine-focus sealed tube	6599 reflections with *I* > 2σ(*I*)
Graphite monochromator	*R*_int_ = 0.029
φ and ω scans	θ_max_ = 31.3°, θ_min_ = 1.5°
Absorption correction: multi-scan (*SADABS*; Bruker, 2009)	*h* = −20→20
*T*_min_ = 0.350, *T*_max_ = 0.696	*k* = −16→15
34224 measured reflections	*l* = −25→25

### Refinement

**Table:**

Refinement on *F*^2^	Primary atom site location: structure-invariant direct methods
Least-squares matrix: full	Secondary atom site location: difference Fourier map
*R*[*F*^2^ > 2σ(*F*^2^)] = 0.031	Hydrogen site location: inferred from neighbouring sites
*wR*(*F*^2^) = 0.067	H-atom parameters constrained
*S* = 1.02	*w* = 1/[σ^2^(*F*_o_^2^) + (0.0274*P*)^2^ + 0.9931*P*] where *P* = (*F*_o_^2^ + 2*F*_c_^2^)/3
8749 reflections	(Δ/σ)_max_ = 0.002
307 parameters	Δρ_max_ = 0.64 e Å^−^^3^
0 restraints	Δρ_min_ = −0.48 e Å^−^^3^

### Special details

**Table:**

Experimental. The crystal was placed in the cold stream of an Oxford Cryosystems Cobra open-flow nitrogen cryostat (Cosier & Glazer, 1986) operating at 100.0 (1) K.
Geometry. All e.s.d.'s (except the e.s.d. in the dihedral angle between two l.s. planes) are estimated using the full covariance matrix. The cell e.s.d.'s are taken into account individually in the estimation of e.s.d.'s in distances, angles and torsion angles; correlations between e.s.d.'s in cell parameters are only used when they are defined by crystal symmetry. An approximate (isotropic) treatment of cell e.s.d.'s is used for estimating e.s.d.'s involving l.s. planes.
Refinement. Refinement of *F*^2^ against ALL reflections. The weighted *R*-factor *wR* and goodness of fit *S* are based on *F*^2^, conventional *R*-factors *R* are based on *F*, with *F* set to zero for negative *F*^2^. The threshold expression of *F*^2^ > σ(*F*^2^) is used only for calculating *R*-factors(gt) *etc*. and is not relevant to the choice of reflections for refinement. *R*-factors based on *F*^2^ are statistically about twice as large as those based on *F*, and *R*- factors based on ALL data will be even larger.

### Fractional atomic coordinates and isotropic or equivalent isotropic displacement parameters (Å^2^)

**Table:**

	*x*	*y*	*z*	*U*_iso_*/*U*_eq_	
Br1A	1.094519 (14)	−0.172320 (16)	0.342402 (11)	0.02211 (5)	
Cl1A	0.47347 (4)	0.16991 (5)	0.02259 (3)	0.02967 (11)	
S1A	1.03184 (3)	−0.00037 (4)	0.16098 (2)	0.01644 (9)	
O1A	0.79290 (8)	0.11587 (11)	0.26956 (7)	0.0185 (3)	
N1A	0.98548 (10)	0.07140 (12)	0.28866 (8)	0.0140 (3)	
H1NA	0.9419	0.0984	0.3077	0.017*	
N2A	0.85365 (10)	0.03620 (12)	0.17480 (8)	0.0139 (3)	
H2NA	0.8370	0.0175	0.1262	0.017*	
C1A	1.12840 (13)	0.19682 (16)	0.35152 (11)	0.0179 (4)	
H1AA	1.0881	0.2654	0.3337	0.022*	
C2A	1.22766 (14)	0.20975 (17)	0.39607 (11)	0.0216 (4)	
H2AA	1.2549	0.2874	0.4092	0.026*	
C3A	1.28731 (13)	0.11006 (18)	0.42148 (11)	0.0230 (4)	
H3AA	1.3554	0.1196	0.4510	0.028*	
C4A	1.24741 (13)	−0.00344 (17)	0.40372 (11)	0.0198 (4)	
H4AA	1.2881	−0.0719	0.4209	0.024*	
C5A	1.14793 (13)	−0.01648 (15)	0.36076 (10)	0.0156 (3)	
C6A	1.08826 (12)	0.08285 (16)	0.33317 (10)	0.0144 (3)	
C7A	0.95520 (12)	0.03821 (15)	0.21252 (10)	0.0135 (3)	
C8A	0.77776 (12)	0.07468 (15)	0.20308 (10)	0.0148 (3)	
C9A	0.67578 (12)	0.05965 (17)	0.14407 (10)	0.0187 (4)	
H9AA	0.6614	−0.0266	0.1349	0.022*	
H9AB	0.6750	0.0960	0.0930	0.022*	
C10A	0.59501 (12)	0.11665 (17)	0.17189 (10)	0.0181 (4)	
H10A	0.5990	0.0843	0.2248	0.022*	
H10B	0.6071	0.2038	0.1776	0.022*	
C11A	0.49134 (13)	0.09517 (18)	0.11575 (11)	0.0215 (4)	
H11A	0.4417	0.1246	0.1403	0.026*	
H11B	0.4807	0.0083	0.1064	0.026*	
Br1B	0.616014 (14)	0.168367 (17)	0.413043 (11)	0.02241 (5)	
Cl1B	1.23151 (3)	0.18536 (4)	0.70479 (3)	0.02501 (10)	
S1B	0.76428 (3)	0.49774 (4)	0.47511 (2)	0.01627 (9)	
O1B	0.93871 (9)	0.18019 (11)	0.42755 (7)	0.0190 (3)	
N1B	0.78048 (10)	0.32012 (13)	0.38055 (8)	0.0152 (3)	
H1NB	0.8114	0.2595	0.3726	0.018*	
N2B	0.91577 (10)	0.34933 (12)	0.49181 (8)	0.0132 (3)	
H2NB	0.9403	0.3934	0.5313	0.016*	
C1B	0.67353 (13)	0.43193 (17)	0.26902 (11)	0.0199 (4)	
H1BA	0.7301	0.4736	0.2647	0.024*	
C2B	0.58029 (13)	0.45596 (17)	0.21637 (11)	0.0231 (4)	
H2BA	0.5731	0.5139	0.1758	0.028*	
C3B	0.49745 (13)	0.39537 (18)	0.22292 (11)	0.0227 (4)	
H3BA	0.4337	0.4121	0.1867	0.027*	
C4B	0.50693 (13)	0.31080 (17)	0.28186 (11)	0.0206 (4)	
H4BA	0.4502	0.2700	0.2867	0.025*	
C5B	0.60057 (13)	0.28675 (16)	0.33370 (10)	0.0169 (3)	
C6B	0.68418 (12)	0.34721 (15)	0.32795 (10)	0.0154 (3)	
C7B	0.82121 (12)	0.38288 (15)	0.44613 (10)	0.0125 (3)	
C8B	0.97074 (12)	0.25286 (16)	0.48090 (10)	0.0147 (3)	
C9B	1.07364 (12)	0.24650 (16)	0.53928 (10)	0.0161 (3)	
H9BA	1.0731	0.2844	0.5896	0.019*	
H9BB	1.1197	0.2927	0.5182	0.019*	
C10B	1.11242 (12)	0.11951 (16)	0.55635 (11)	0.0180 (4)	
H10C	1.0698	0.0750	0.5817	0.022*	
H10D	1.1082	0.0792	0.5057	0.022*	
C11B	1.21905 (13)	0.11609 (19)	0.61013 (11)	0.0227 (4)	
H11C	1.2622	0.1582	0.5841	0.027*	
H11D	1.2416	0.0320	0.6187	0.027*	

### Atomic displacement parameters (Å^2^)

**Table:**

	*U*^11^	*U*^22^	*U*^33^	*U*^12^	*U*^13^	*U*^23^
Br1A	0.02895 (10)	0.01320 (9)	0.02297 (10)	0.00066 (8)	0.00623 (8)	0.00138 (7)
Cl1A	0.0230 (2)	0.0355 (3)	0.0255 (2)	0.0028 (2)	0.00004 (19)	0.0106 (2)
S1A	0.01481 (19)	0.0212 (2)	0.01338 (19)	0.00319 (17)	0.00442 (16)	0.00091 (17)
O1A	0.0152 (6)	0.0234 (7)	0.0159 (6)	0.0014 (5)	0.0035 (5)	−0.0053 (5)
N1A	0.0116 (6)	0.0157 (7)	0.0147 (7)	0.0013 (6)	0.0040 (5)	−0.0016 (6)
N2A	0.0133 (6)	0.0159 (7)	0.0111 (6)	0.0008 (6)	0.0015 (5)	−0.0018 (5)
C1A	0.0182 (8)	0.0184 (9)	0.0174 (9)	−0.0011 (7)	0.0056 (7)	−0.0015 (7)
C2A	0.0210 (9)	0.0218 (9)	0.0205 (9)	−0.0050 (8)	0.0044 (7)	−0.0039 (7)
C3A	0.0147 (8)	0.0321 (11)	0.0196 (9)	0.0008 (8)	0.0013 (7)	−0.0022 (8)
C4A	0.0183 (8)	0.0232 (10)	0.0166 (8)	0.0053 (8)	0.0032 (7)	0.0010 (7)
C5A	0.0189 (8)	0.0138 (8)	0.0141 (8)	0.0003 (7)	0.0049 (7)	0.0002 (6)
C6A	0.0128 (7)	0.0182 (9)	0.0118 (8)	0.0009 (7)	0.0029 (6)	0.0005 (6)
C7A	0.0149 (8)	0.0111 (8)	0.0136 (8)	0.0013 (6)	0.0030 (6)	0.0022 (6)
C8A	0.0141 (7)	0.0138 (8)	0.0157 (8)	0.0016 (7)	0.0032 (6)	0.0002 (6)
C9A	0.0135 (8)	0.0244 (10)	0.0160 (8)	0.0015 (7)	0.0011 (7)	−0.0044 (7)
C10A	0.0141 (8)	0.0233 (10)	0.0162 (8)	−0.0013 (7)	0.0036 (7)	−0.0019 (7)
C11A	0.0158 (8)	0.0290 (10)	0.0193 (9)	−0.0016 (8)	0.0048 (7)	0.0014 (8)
Br1B	0.02251 (9)	0.02434 (10)	0.02018 (9)	−0.00204 (8)	0.00630 (7)	0.00036 (8)
Cl1B	0.0219 (2)	0.0316 (3)	0.0171 (2)	0.00387 (19)	−0.00056 (17)	−0.00221 (19)
S1B	0.01620 (19)	0.0171 (2)	0.01410 (19)	0.00422 (17)	0.00255 (16)	−0.00142 (16)
O1B	0.0156 (6)	0.0193 (7)	0.0187 (6)	0.0034 (5)	0.0004 (5)	−0.0053 (5)
N1B	0.0119 (6)	0.0161 (7)	0.0153 (7)	0.0037 (6)	0.0009 (5)	−0.0024 (6)
N2B	0.0122 (6)	0.0138 (7)	0.0118 (6)	0.0007 (5)	0.0011 (5)	−0.0022 (5)
C1B	0.0158 (8)	0.0222 (10)	0.0200 (9)	0.0011 (7)	0.0029 (7)	0.0003 (7)
C2B	0.0221 (9)	0.0233 (10)	0.0190 (9)	0.0064 (8)	−0.0008 (7)	0.0039 (8)
C3B	0.0146 (8)	0.0274 (11)	0.0214 (9)	0.0052 (8)	−0.0012 (7)	−0.0041 (8)
C4B	0.0139 (8)	0.0238 (10)	0.0229 (9)	−0.0001 (7)	0.0040 (7)	−0.0071 (8)
C5B	0.0177 (8)	0.0173 (9)	0.0155 (8)	0.0020 (7)	0.0051 (7)	−0.0041 (7)
C6B	0.0125 (7)	0.0178 (9)	0.0137 (8)	0.0030 (7)	0.0008 (6)	−0.0044 (6)
C7B	0.0126 (7)	0.0134 (8)	0.0115 (7)	−0.0008 (6)	0.0038 (6)	0.0025 (6)
C8B	0.0131 (7)	0.0156 (9)	0.0149 (8)	0.0005 (7)	0.0034 (6)	0.0005 (6)
C9B	0.0116 (7)	0.0175 (9)	0.0171 (8)	0.0007 (7)	0.0013 (6)	−0.0010 (7)
C10B	0.0156 (8)	0.0200 (9)	0.0157 (8)	0.0030 (7)	0.0009 (7)	−0.0025 (7)
C11B	0.0199 (9)	0.0292 (11)	0.0176 (9)	0.0088 (8)	0.0040 (7)	−0.0017 (8)

### Geometric parameters (Å, º)

**Table:**

Br1A—C5A	1.8889 (17)	Br1B—C5B	1.8955 (18)
Cl1A—C11A	1.7983 (19)	Cl1B—C11B	1.8071 (19)
S1A—C7A	1.6751 (17)	S1B—C7B	1.6792 (17)
O1A—C8A	1.221 (2)	O1B—C8B	1.224 (2)
N1A—C7A	1.337 (2)	N1B—C7B	1.328 (2)
N1A—C6A	1.430 (2)	N1B—C6B	1.426 (2)
N1A—H1NA	0.8445	N1B—H1NB	0.8421
N2A—C7A	1.385 (2)	N2B—C8B	1.379 (2)
N2A—C8A	1.386 (2)	N2B—C7B	1.384 (2)
N2A—H2NA	0.8465	N2B—H2NB	0.8391
C1A—C2A	1.390 (2)	C1B—C6B	1.384 (2)
C1A—C6A	1.394 (2)	C1B—C2B	1.387 (2)
C1A—H1AA	0.9500	C1B—H1BA	0.9500
C2A—C3A	1.388 (3)	C2B—C3B	1.389 (3)
C2A—H2AA	0.9500	C2B—H2BA	0.9500
C3A—C4A	1.386 (3)	C3B—C4B	1.385 (3)
C3A—H3AA	0.9500	C3B—H3BA	0.9500
C4A—C5A	1.385 (2)	C4B—C5B	1.386 (2)
C4A—H4AA	0.9500	C4B—H4BA	0.9500
C5A—C6A	1.390 (2)	C5B—C6B	1.393 (2)
C8A—C9A	1.507 (2)	C8B—C9B	1.505 (2)
C9A—C10A	1.517 (2)	C9B—C10B	1.520 (2)
C9A—H9AA	0.9900	C9B—H9BA	0.9900
C9A—H9AB	0.9900	C9B—H9BB	0.9900
C10A—C11A	1.516 (2)	C10B—C11B	1.516 (2)
C10A—H10A	0.9900	C10B—H10C	0.9900
C10A—H10B	0.9900	C10B—H10D	0.9900
C11A—H11A	0.9900	C11B—H11C	0.9900
C11A—H11B	0.9900	C11B—H11D	0.9900
			
C7A—N1A—C6A	122.48 (14)	C7B—N1B—C6B	121.93 (14)
C7A—N1A—H1NA	117.2	C7B—N1B—H1NB	117.8
C6A—N1A—H1NA	119.3	C6B—N1B—H1NB	120.1
C7A—N2A—C8A	128.57 (14)	C8B—N2B—C7B	127.96 (14)
C7A—N2A—H2NA	114.6	C8B—N2B—H2NB	118.1
C8A—N2A—H2NA	116.3	C7B—N2B—H2NB	113.9
C2A—C1A—C6A	119.70 (17)	C6B—C1B—C2B	120.03 (17)
C2A—C1A—H1AA	120.2	C6B—C1B—H1BA	120.0
C6A—C1A—H1AA	120.2	C2B—C1B—H1BA	120.0
C3A—C2A—C1A	120.46 (18)	C1B—C2B—C3B	120.01 (18)
C3A—C2A—H2AA	119.8	C1B—C2B—H2BA	120.0
C1A—C2A—H2AA	119.8	C3B—C2B—H2BA	120.0
C4A—C3A—C2A	119.96 (17)	C4B—C3B—C2B	120.57 (16)
C4A—C3A—H3AA	120.0	C4B—C3B—H3BA	119.7
C2A—C3A—H3AA	120.0	C2B—C3B—H3BA	119.7
C5A—C4A—C3A	119.64 (17)	C3B—C4B—C5B	118.91 (17)
C5A—C4A—H4AA	120.2	C3B—C4B—H4BA	120.5
C3A—C4A—H4AA	120.2	C5B—C4B—H4BA	120.5
C4A—C5A—C6A	120.81 (16)	C4B—C5B—C6B	121.06 (17)
C4A—C5A—Br1A	118.44 (13)	C4B—C5B—Br1B	119.73 (14)
C6A—C5A—Br1A	120.73 (13)	C6B—C5B—Br1B	119.20 (13)
C5A—C6A—C1A	119.39 (15)	C1B—C6B—C5B	119.40 (15)
C5A—C6A—N1A	121.72 (15)	C1B—C6B—N1B	119.93 (15)
C1A—C6A—N1A	118.85 (15)	C5B—C6B—N1B	120.64 (16)
N1A—C7A—N2A	116.99 (15)	N1B—C7B—N2B	116.64 (15)
N1A—C7A—S1A	124.24 (13)	N1B—C7B—S1B	123.60 (13)
N2A—C7A—S1A	118.77 (12)	N2B—C7B—S1B	119.76 (12)
O1A—C8A—N2A	122.79 (15)	O1B—C8B—N2B	122.61 (15)
O1A—C8A—C9A	123.88 (15)	O1B—C8B—C9B	123.30 (15)
N2A—C8A—C9A	113.33 (14)	N2B—C8B—C9B	114.08 (14)
C8A—C9A—C10A	112.48 (14)	C8B—C9B—C10B	113.21 (14)
C8A—C9A—H9AA	109.1	C8B—C9B—H9BA	108.9
C10A—C9A—H9AA	109.1	C10B—C9B—H9BA	108.9
C8A—C9A—H9AB	109.1	C8B—C9B—H9BB	108.9
C10A—C9A—H9AB	109.1	C10B—C9B—H9BB	108.9
H9AA—C9A—H9AB	107.8	H9BA—C9B—H9BB	107.8
C11A—C10A—C9A	113.10 (15)	C11B—C10B—C9B	112.12 (15)
C11A—C10A—H10A	109.0	C11B—C10B—H10C	109.2
C9A—C10A—H10A	109.0	C9B—C10B—H10C	109.2
C11A—C10A—H10B	109.0	C11B—C10B—H10D	109.2
C9A—C10A—H10B	109.0	C9B—C10B—H10D	109.2
H10A—C10A—H10B	107.8	H10C—C10B—H10D	107.9
C10A—C11A—Cl1A	111.27 (13)	C10B—C11B—Cl1B	111.50 (12)
C10A—C11A—H11A	109.4	C10B—C11B—H11C	109.3
Cl1A—C11A—H11A	109.4	Cl1B—C11B—H11C	109.3
C10A—C11A—H11B	109.4	C10B—C11B—H11D	109.3
Cl1A—C11A—H11B	109.4	Cl1B—C11B—H11D	109.3
H11A—C11A—H11B	108.0	H11C—C11B—H11D	108.0
			
C6A—C1A—C2A—C3A	0.9 (3)	C6B—C1B—C2B—C3B	0.3 (3)
C1A—C2A—C3A—C4A	−1.2 (3)	C1B—C2B—C3B—C4B	0.1 (3)
C2A—C3A—C4A—C5A	−0.1 (3)	C2B—C3B—C4B—C5B	−0.6 (3)
C3A—C4A—C5A—C6A	1.9 (3)	C3B—C4B—C5B—C6B	0.9 (3)
C3A—C4A—C5A—Br1A	−176.78 (14)	C3B—C4B—C5B—Br1B	−178.30 (14)
C4A—C5A—C6A—C1A	−2.3 (3)	C2B—C1B—C6B—C5B	0.0 (3)
Br1A—C5A—C6A—C1A	176.36 (13)	C2B—C1B—C6B—N1B	178.15 (16)
C4A—C5A—C6A—N1A	179.92 (16)	C4B—C5B—C6B—C1B	−0.6 (3)
Br1A—C5A—C6A—N1A	−1.4 (2)	Br1B—C5B—C6B—C1B	178.63 (13)
C2A—C1A—C6A—C5A	0.9 (3)	C4B—C5B—C6B—N1B	−178.72 (16)
C2A—C1A—C6A—N1A	178.74 (16)	Br1B—C5B—C6B—N1B	0.5 (2)
C7A—N1A—C6A—C5A	−75.6 (2)	C7B—N1B—C6B—C1B	86.2 (2)
C7A—N1A—C6A—C1A	106.65 (19)	C7B—N1B—C6B—C5B	−95.7 (2)
C6A—N1A—C7A—N2A	−176.84 (14)	C6B—N1B—C7B—N2B	−178.95 (14)
C6A—N1A—C7A—S1A	3.1 (2)	C6B—N1B—C7B—S1B	1.1 (2)
C8A—N2A—C7A—N1A	6.6 (3)	C8B—N2B—C7B—N1B	−5.2 (2)
C8A—N2A—C7A—S1A	−173.40 (14)	C8B—N2B—C7B—S1B	174.71 (13)
C7A—N2A—C8A—O1A	−1.3 (3)	C7B—N2B—C8B—O1B	−2.2 (3)
C7A—N2A—C8A—C9A	179.18 (16)	C7B—N2B—C8B—C9B	176.77 (15)
O1A—C8A—C9A—C10A	7.8 (3)	O1B—C8B—C9B—C10B	−30.9 (2)
N2A—C8A—C9A—C10A	−172.60 (15)	N2B—C8B—C9B—C10B	150.22 (15)
C8A—C9A—C10A—C11A	−176.15 (15)	C8B—C9B—C10B—C11B	175.45 (15)
C9A—C10A—C11A—Cl1A	−66.74 (19)	C9B—C10B—C11B—Cl1B	60.18 (19)

### Hydrogen-bond geometry (Å, º)

**Table:**

*D*—H···*A*	*D*—H	H···*A*	*D*···*A*	*D*—H···*A*
N1*A*—H1*NA*···O1*A*	0.84	2.01	2.6847 (19)	136
N1*B*—H1*NB*···O1*B*	0.84	1.97	2.6464 (19)	136
N1*A*—H1*NA*···O1*B*	0.84	2.33	2.9976 (18)	137
N1*B*—H1*NB*···O1*A*	0.84	2.39	3.0566 (19)	137
N2*A*—H2*NA*···S1*B*^i^	0.85	2.56	3.3931 (15)	168
N2*B*—H2*NB*···S1*A*^ii^	0.84	2.56	3.3928 (14)	171
C9*B*—H9*BA*···S1*A*^ii^	0.99	2.87	3.7237 (18)	145
C9*B*—H9*BB*···S1*B*^iii^	0.99	2.84	3.7248 (18)	149

Symmetry codes: (i) *x*, −*y*+1/2, *z*−1/2; (ii) *x*, −*y*+1/2, *z*+1/2; (iii) −*x*+2, −*y*+1, −*z*+1.
